# Aneurysmal bone cyst D4-D5 level

**DOI:** 10.11604/pamj.2022.42.280.36079

**Published:** 2022-08-15

**Authors:** Snehal Subrat Samal, Subrat Narendra Samal

**Affiliations:** 1 Department of Neurophysiotherapy, Ravi Nair Physiotherapy College, Datta Meghe Institute of Medical Sciences, Sawangi, Meghe, Wardha, Maharashtra, India,; 2 Department of Musculoskeletal Physiotherapy, Datta Meghe College of Physiotherapy, Wanadongri, Nagpur, Maharashtra, India

**Keywords:** Aneurysmal cyst, spinal tumor, multiocular cyst

## Image in medicine

Presenting a magnetic resonance image (MRI) finding of an 11-year-old female child who presented to us with the chief complaints of pain at thoracic spine and bilateral lower limbs. Magnetic resonance findings as per imaging protocol (sagittal TSE T2W1) revealed a multiocular cystic lesion which is noted in D5 vertebral body extended into both lamina and spinous process showing fluid-fluid level compressing spinal cord at D4 and D5 level likely s/o aneurysmal cyst. Aneurysmal cyst is rare. In 16% of cases it commonly affects the spine. The patient narrated that the weakness rapidly increased within 4 days and she started walking with support. For these complaints, the parents took her to a private practitioner where nerve conduction velocity (NCV) was done which was normal. The patients had more weakness at right lower limb as compared to left lower limb. She was admitted for the same and post-surgery after 3 days´ physiotherapy had been started. Post-operative physiotherapy assessment shows decreased superficial sensations, reflexes and strength (MMT grade 3/5 at right lower limb and grade 4/5 at left lower limb). Strengthening exercises of lower limb had started along with bedside mobilization. Before discharge, she had a grade of 4+ on the muscle power assessment (MRC) grading scale. Early medical and surgical management along with post-operative rehabilitation is the way to recovery in such patients.

**Figure 1:**
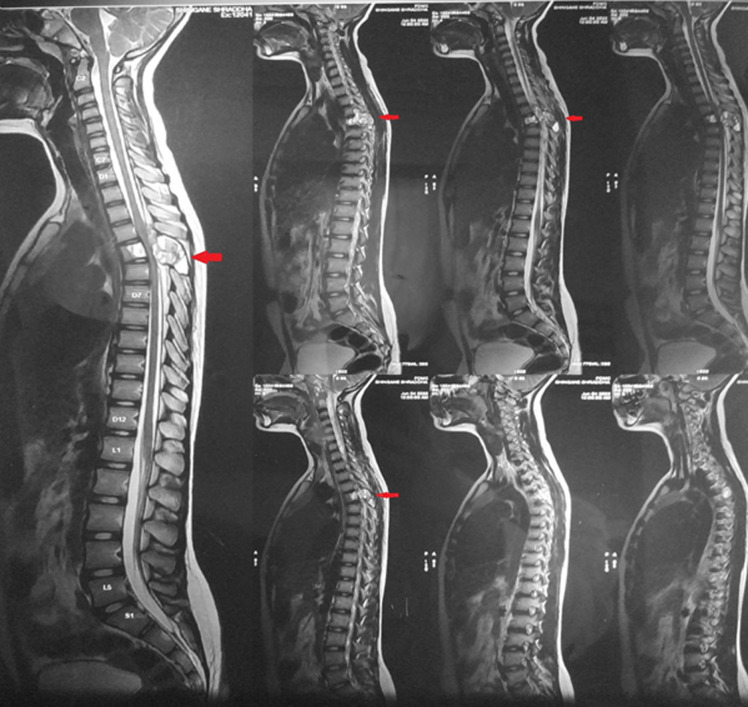
MRI finding of aneurysmal bone cyst at D4-D5 level

